# A response to information criterion-based clustering with order-restricted candidate profiles in short time-course microarray experiments

**DOI:** 10.1186/1471-2105-10-438

**Published:** 2009-12-22

**Authors:** Shyamal D Peddada, David M Umbach, Shawn F Harris

**Affiliations:** 1 Biostatistics Branch, National Institute of Environmental Health Sciences Research Triangle Park, NC 27709, USA; 2 SRA International Inc, Durham, NC 27713,USA

## Abstract

**Background:**

For gene expression data obtained from a time-course microarray experiment, Liu et al. [1] developed a new algorithm for clustering genes with similar expression profiles over time. Performance of their proposal was compared with three other methods including the order-restricted inference based methodology of Peddada et al. [2,3]. In this note we point out several inaccuracies in Liu et al. [1] and conclude that the order-restricted inference based methodology of Peddada et al. (programmed in the software ORIOGEN) indeed operates at the desired nominal Type 1 error level, an important feature of a statistical decision rule, while being computationally substantially faster than indicated by Liu et al. [1].

**Results:**

Application of ORIOGEN to the well-known breast cancer cell line data of Lobenhofer et al. [4] revealed that ORIOGEN software took only 21 minutes to run (using 100,000 bootstraps with p = 0.0025), substantially faster than the 72 hours found by Liu et al. [1] using Matlab. Also, based on a data simulated according to the model and parameters of simulation 1 (*σ*^2 ^= 1, *M *= 5) in [1] we found that ORIOGEN took less than 30 seconds to run in stark contrast to Liu et al. who reported that their implementation of the same algorithm in R took 2979.29 seconds. Furthermore, for the simulation studies reported in [1], unlike the claims made by Liu et al. [1], ORIOGEN always maintained the desired false positive rate. According to Figure three in Liu et al. [1] their algorithm had a false positive rate ranging approximately from 0.20 to 0.70 for the scenarios that they simulated.

**Conclusions:**

Our comparisons of run times indicate that the implementations of ORIOGEN's algorithm in Matlab and R by Liu et al. [1] is inefficient compared to the publicly available JAVA implementation. Our results on the false positive rate of ORIOGEN suggest some error in Figure three of Liu et al. [1], perhaps due to a programming error.

## Background

A short-series time-course microarray experiment induces a natural constraint on the mean expression of a gene or a probe over time. Thus one may expect a systematic pattern to the mean expression of a gene as long as the time points are not too far apart to lose the biological relevance of a time-course experiment. For example, for some genes the mean expression may monotonically increase (or decrease) over time, whereas some others may display an (inverted) umbrella shaped pattern, etc. Although, one may consider using a parametric model to describe the pattern of expression across time points, a simple nonparametric approach can be used to express the pattern of expression across time using mathematical inequalities (known as order restrictions). This strategy was first exploited in [2] and subsequently software called ORIOGEN (Ordered Restricted Inference for Ordered Gene ExpressioN) was developed ([3]). It has been publicly available at ORIOGEN - Order Restricted Inference for Ordered Gene ExpressioN http://www.niehs.nih.gov/research/resources/software/oriogen/index.cfm since 2005, and an upgraded version (2.2.1) has been available since February 1, 2007.

Liu et al. [1] introduce an interesting alternative methodology for clustering genes using order-restricted inference methodology. Their strategy differs from that of [2,3] in that they avoid the bootstrap computation of p-value for identifying significant genes. Instead, they use an information theoretic model selection criterion to assign genes to temporal patterns (clusters). Once genes are clustered, they evaluate the reliability of each cluster using a bootstrap algorithm along the lines of Kerr and Churchill [5]. Liu et al. [1] make several statements about our methodology [2,3] that are erroneous and require clarification.

The methodology of Peddada et al. [2], implemented in ORIOGEN [3], controls the Type I error rate (false positive rate) at any pre-specified level. The methodology of Liu et al. [1], implemented in ORICC, does not control Type I error rate explicitly. It thereby risks attributing differential expression through time to an excessive proportion of genes whose expression does not truly change. Given the large multiple testing problem inherent in microarray analyses (*e.g*., the Human Affymetrix chip has ~45,000 probes), use of procedures that control Type I error, or a related quantity like the false discovery rate (FDR), is crucial.

## Results

### Computation time

Throughout their paper, Liu et al. [1] claim that Peddada et al. methodology is excessively computationally intensive. The many bootstrap samples needed for precise estimation of small p values is indeed computationally demanding; but the run times mentioned by Liu et al. for our methodology seemed extremely long in our experience. On page 2 they assert that their implementation of "Peddada's method" required 72 hours to analyze the breast cancer cell-line data of [4]. They do not state how many bootstrap samples they used and what p-value they used in their analysis. Although they cite our publicly available software ORIOGEN, they appear to have written their own code rather than using ORIOGEN. They state that they implemented our methodology (calling it "Peddada's methodology") in Matlab (page 2) and again in R (page 5). Their performance estimates were not based on the ORIOGEN software that is freely available from our website. We were surprised that the authors made the effort to re-code our algorithm in two different languages when our software is available without charge.

To examine their claims about run times, we implemented ORIOGEN on the breast cancer cell-line data of [4], using 100,000 bootstraps, with a p-value of 0.0025. We found that ORIOGEN took only 21 minutes to run, not 72 hours as stated in [1] for their Matlab implementation. We also used ORIOGEN to analyze data simulated exactly as described on pages 5-6 of [1]. The authors claim on page 7 that their implementation of "Peddada's method" in R took 2979.29 seconds to analyze the simulated data when *σ*^2 ^= 1 and *M *= 5; however, for the same simulation conditions, ORIOGEN took about 30 seconds to run. For these analyses, we employed a Dell desktop PC with an Intel Xeon CPU 2.33 GHz with 3.00 GB of RAM. ORIOGEN was developed by two professional computer programmers who tested it repeatedly before making it public. These exceptionally large discrepancies in run times between ORIOGEN and Liu et al.'s implementation of its algorithm lead us to conclude that either Liu et al. either misinterpreted details of our methodology or their coding of it is extremely inefficient.

### False positive rates

In Figure three, Liu et al. [1] compare the false positive rate of our method with theirs. They claim to have run our method at a level of significance of 0.025 (for each simulated gene) using 200 bootstraps (page 6). In their simulation study they consider 200 "null" genes and 2000 "non-null" genes and define false positive rate to be the proportion of null genes that are declared significant. Based on their Figure three, they report that our method can have a false positive rate as high as 0.50 at the nominal rate of 0.025. This result is incorrect. We generated the 2200 genes according to the patterns described by the authors on page 9 which included 200 "null genes". Exactly as in [1], we implemented ORIOGEN with a level of significance of 0.025 and 200 bootstraps for the 6 patterns of *σ*^2 ^and as many replicates as in [1]. We found that ORIOGEN always performed at the desired nominal level of 0.025, as it was designed to do. It appears that Liu et al. [1] misinterpreted our methodology, made some programming errors in coding it, or miscalculated/misreported the false positive rates.

Most statisticians and bioinformaticians recognize that maintaining a pre-specified false positive rate is an important requirement for statistical testing procedures. In fact, scientists want to avoid reporting an excessive number of genes as differentially expressed when they are not. During the past decade, much research has been devoted to developing sound methods for controlling false discovery rates in gene expression microarray studies. In contrast to ORIOGEN, which maintains the nominal false positive rate and provides estimates of q-values often used to control false discovery, ORICC, the method proposed by Liu et al., does not control the false positive rate or the false discovery rate at a pre-specified level. Consequently, ORICC can sometimes have an unusually high false positive rate, as high as 0.70 according to Figure three of [1]. In the bottom right panel of Figure three of [1], the lowest false positive rate that ORICC achieved for the simulated data is 0.20.

### Other erroneous statements

(a) Liu et al. make an incorrect assertion regarding the universal domination property of order-restricted maximum likelihood estimator (MLE) (page 14 of [1]). They assert that the order-restricted MLE universally dominates the unrestricted MLE and wrongly attribute this theoretical property to Hwang and Peddada [6]. Hwang and Peddada did not prove the result in the generality stated in [1]; they proved it only for independently normally distributed data when the means satisfy a monotone order. On the contrary, the main emphasis of [6] is to demonstrate that the order-restricted MLE may actually perform poorly under certain conditions. Hence in [6] Hwang and Peddada introduced an alternative to the order-restricted MLE for estimation of parameters satisfying constraints. ORIOGEN uses this new estimation procedure instead of the order-restricted MLE. When the order restriction is monotonic, the two procedures coincide.

(b) Liu et al. also state incorrectly on page 5 that "Peddada's method then carried out a bootstrap-based likelihood ratio test". Because ORIOGEN is not working with order-restricted MLE's to begin with, there is no explicit likelihood with which to construct a likelihood-based test. Consequently, its test statistic is more along the lines of a "Wald" type statistic and not a likelihood ratio. In the same sentence as stated above, on page 5, Liu et al. suggest that Peddada et al. use the bootstrap-likelihood ratio test to decide a gene's best matched profile. This statement is not correct. Peddada et al. used the bootstrap to select significant genes but assigned genes to profiles using a goodness-of-fit criterion.

### A Simulation study

In their simulation study, Liu et al. [1] considered 200 "null" (or non-differentially expressed) genes and 2000 "non-null" (differentially expressed/true positive) genes. Thus at most 10% are non-differentially expressed whereas the overwhelming majority, about 90%, are differentially expressed. If this were the true nature of the data, then a biologist may want to skip any formal statistical methodology and take all 2200 genes - this selection rule will assure him/her 100% discovery of true genes at a small price of at most 10% false discovery rate (FDR). From our experience, it would be more realistic to expect that most genes in a microarray study would be non-differentially expressed.

#### Study design

In this simulation study we almost mimicked the simulation experiment of Liu et al. [1] with the major exception that we considered 12000 null (or non-differentially expressed) genes and 4000 non-null (true positives) genes. Thus, 25% are true positives and about 75% are true nulls.

We generated our data according to the model and the parameters used in Liu et al.[1] except that we have more null genes than non-null genes as commonly observed in gene expression studies:

In the above model, for a gene *g*, *g *= 1,2,...,16000,  is the observed expression of the *j*^*th *^replicate, *j *= 1,2,...,8, in the *i*^*th *^treatment group, *i *= 1,2,...,6, and  is the true mean expression of *g*^*th *^gene in the *i*^*th *^treatment group. We considered 2 patterns of variance *σ*^2^(= 0.2,1.)' within each pattern of  described below.

**Pattern 1: **(Null) *μ*^*g *^=(0,0,0,0,0,0)' - 6000 samples corresponding to each variance pattern, and hence 12000 null genes.

**Pattern 2: **(Increasing) *μ *= (0,0.5,1,1.5,2,2.5)' - 200 samples corresponding to each variance pattern, and hence 400 increasing genes.

**Pattern 3: **(Decreasing) *μ *= (0,-0.5,-1,-1.5,-2,-2.5)' - 200 samples corresponding to each variance pattern, and hence 400 decreasing genes.

**Pattern 4: **(Umbrella Peak at 2) *μ *= (0,0.5,0,-0.5,-1,-1.5)' - 200 samples corresponding to each variance pattern, and hence 400 umbrella pattern genes.

**Pattern 5: **(Inverted Umbrella Min at 2) *μ *= (0,-0.5,0,0.5,1,1.5)' - 200 samples corresponding to each variance pattern, and hence 400 inverted umbrella pattern genes.

**Pattern 6: **(Umbrella Peak at 3) *μ *= (0,0.5,1,0.5,0,-0.5)' - 200 samples corresponding to each variance pattern, and hence 400 umbrella pattern genes.

**Pattern 7: **(Inverted Umbrella Min at 3) *μ *= (0,-0.5,-1,-0.5,0,0.5)' - 200 samples corresponding to each variance pattern, and hence 400 inverted umbrella pattern genes.

**Pattern 8: **(Umbrella Peak at 4) *μ *= (0,0.5,1,1.5,1,0.5)' - 200 samples corresponding to each variance pattern, and hence 400 umbrella pattern genes.

**Pattern 9: **(Inverted Umbrella Min at 4) *μ *= (0,-0.5,-1,-1.5,-1,-0.5)' - 200 samples corresponding to each variance pattern, and hence 400 inverted umbrella pattern genes.

**Pattern 10: **(Umbrella Peak at 5) *μ *= (0,0.5,1,1.5,2,1.5)' - 200 samples corresponding to each variance pattern, and hence 400 umbrella pattern genes.

**Pattern 11: **(Inverted Umbrella Min at 5) *μ *= (0,-0.5,-1,-1.5,-2,-1.5)' - 200 samples corresponding to each variance pattern, and hence 400 inverted umbrella pattern genes.

Thus the total number of genes considered in this simulation study is 16000 consisting of 12000 null and 4000 non-null.

#### Results

We applied ORICC, by downloading the software from the website provided in [1], and ORIOGEN 2.2.1. We applied ORIOGEN using a p-value cut off (or level of significance) of 0.01 and the reclassification p-value of 0.90 for patterns. Since we are using a cut-off of 0.01, it is sufficient to run ORIOGEN using 10,000 bootstraps. ORICC does not have any such controls. Results of our simulation study are summarized in Table 1.

**Table 1:** Comparison of ORIOGEN and ORICC using a simulated data. (due to Peddada et al.)

Criteria	ORIOGEN	ORICC
Number of nulls selected	95	3559
Number of true positives selected	3335	3957
		
Total number of discoveries	3430	7516
		
Number of discoveries with correct non-null cluster assignment	2917	3322
		
Type I error rate	0.008	0.297
False discovery rate (FDR)	0.028	0.474
		
Power	0.834	0.989
		
Proportion of discoveries with correct non-null cluster assignment	0.729	0.831
		
Proportion of discoveries with correct non-null cluster assignment among the correctly selected non-null genes	0.875	0.840
		
Total error	0.074	0.265

As expected, ORIOGEN performed at the desired Type I error rate (0.008 ~0.01) and consequently provided a better control of FDR than ORICC did. ORICC had an unacceptably high Type I error and FDR (28% and 47%, respectively). Consequently, it is not surprising that ORICC had a higher power (almost 99%) and more correct cluster assignments than ORIOGEN. It is interesting to note, however, that among the correctly identified true positive genes, ORIOGEN did a better job of pattern assignment than ORICC (87.5% vs 84%). Also, the overall error rate (as defined in Liu et al. [1]) for ORICC was almost four times as large as ORIOGEN (26.5% versus 7.4%). We would like to point out we have performed several simulation studies assuming the majority of genes are null and considering various patterns of means and variances; we find qualitatively similar results as above for all of them. One main drawback with the simulation study reported in [1] is that it was too narrow. It did not span a sufficient range for the proportion of null genes in the underlying population and, thereby, led to an overly generous assessment of ORICC's total-error performance. The lower total error of ORICC compared to ORIOGEN as reported in [1] hinges on the proportion of nulls being small (in our view, unrealistically small) in the Liu et al. simulation scenarios.

## Conclusions

In general we agree with the spirit of Liu et al.: there is a great opportunity to use order-restricted inference methodology for analyzing time-course and dose-response studies. It would certainly be important to improve existing methodology and to evaluate the reliability of cluster assignment of genes according to their time-course profile. Such research could prove useful for identifying genes that participate together in various biological processes. We however, conclude that the methodology proposed in [1] can potentially be subject to a very high (a) false positive rate, (b) false discovery rate and (c) overall error rate.

## Authors' contributions

SDP, DMU and SFH contributed equally in writing of this manuscript. SDP and SFH performed the numerical computations. All authors read and approved the final manuscript

## Authors' information

SDP and DMU are members of the Biostatistics Branch, National Institute of Environmental Health Sciences Research Triangle Park, NC 27709. They may be contacted at Peddada@niehs.nih.gov, Umbach@niehs.nih.gov, respectively.

SFH is a Software Engineer at SRA International Inc., Durham, NC 27713 and he may be contacted at Shawn_Harris@sra.com.

## Acknowledgements

The research of SDP and DMU was supported by the Intramural Research Program of the NIH, National Institute of Environmental Health Sciences (Z01 ES101744-04). The authors thank the editors for giving them the opportunity to respond to the article by Liu et al. [1].

## Response to Peddada et al

Tianqing Liu, Nan Lin, Ningzhong Shi and Baoxue Zhang

Corresponding author: Baoxue Zhang

We are delighted to see a thorough correspondence to our paper [7]from Dr. Peddada and his coauthors. In the correspondence, Peddada *et al*. made several comments about our paper on the comparison between their clustering approach, ORIOGEN [8]and our ORICC algorithm in [7].

The first comment is that the computation time reported in [7]about ORIOGEN is not accurate, and it could be we either misinterpreted details of ORIOGEN or our coding is extremely inefficient.

We first need to point out that, in our paper, we compare between ORICC and Peddada's method that refers to the algorithm in [9]. ORIOGEN is based on the algorithm in [9]but with some slight modification. Our experience, in both simulation and real data analysis, suggested that ORIOGEN and the original algorithm in [9]give very similar clustering results. However, the early paper [9]is written in more details and allows our own implementation of the algorithm in Matlab and R. We have carefully examined our coding and found no error.

We believe that the difference between our reported computation time and that in Peddada *et al*.'s correspondence is mainly due to the efficiency of different computer languages. This is because the bootstrap procedure in Peddada's method requires a very large number of iterations, and JAVA is much more efficient than Matlab or R in looping. A fair comparison of the computational efficiency between two methods should be made on the same platform. Therefore, we implemented both methods in R and reported the computation time in our paper. The computation time of Peddada's method reported on page 3 in [7]is based on our early implementation of the method in Matlab and it used 100,000 bootstrap samples. We also tried ORIOGEN (with 100,000 bootstrap samples), implemented in JAVA by Peddada *et al*. [8], on our computer to analyze the breast cancer cell line data in [10], and the computation time was 2877 seconds, whereas it only took ORICC (implemented in R) 15.69 seconds. Our computer is a workstation with a 2.30 GHz AMD Athlon(tm) 64 × 2 Dual Core 4400+processor and a 2.00 GB memory. Note that this breast cancer cell line data has been processed and contains just about 1900 genes. Its size is relatively small compared to most current microarray studies. We further applied ORICC and ORIOGEN (with 100,000 bootstrap samples) to a simulated data set that contains 5500 genes. ORIOGEN was implemented using 100,000 bootstraps, with a p-value of 0.0025. The run time for ORICC and ORIOGEN is 74.03 seconds versus 21,104 seconds. We agree with Peddada *et al*. that the JAVA software ORIOGEN is a very efficient implementation of the algorithm. However, for most microarray studies nowadays involving more than 5,000 genes, without a super powerful computer, it can still take very long for the analyst to obtain the clustering result.

**Figure 1:**
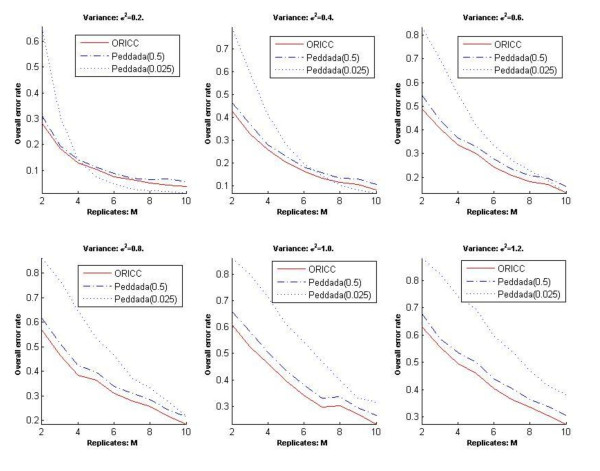
**Simulation 1: The overall error rate of Peddada's method and the one-stage ORICC algorithm**. (due to Liu et al.)

**Figure 2:**
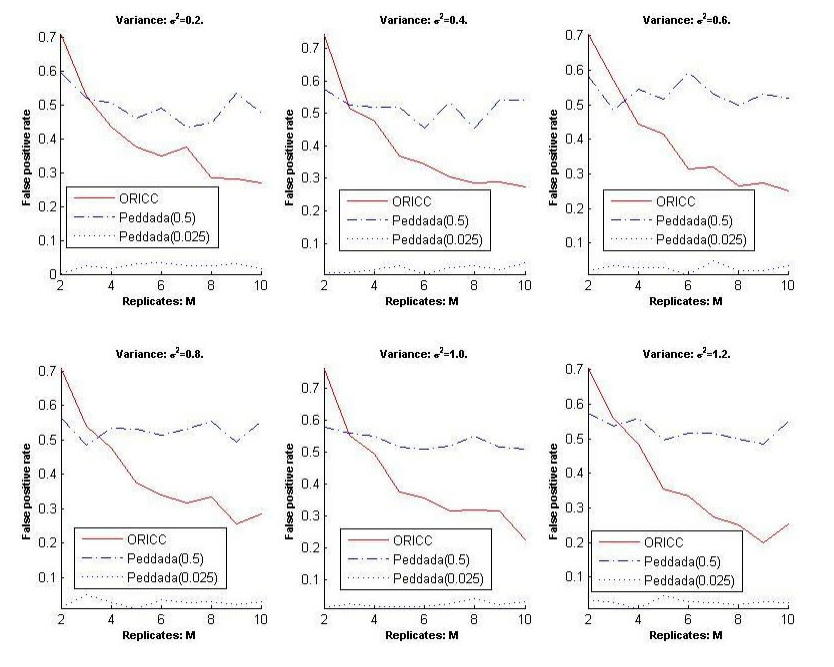
**Simulation 1: The false positive rate of Peddada's method and the one-stage ORICC algorithm**. (due to Liu et al.)

**Figure 3:**
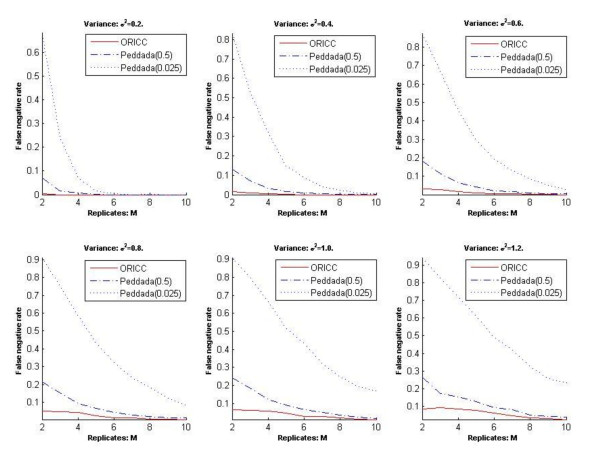
**Simulation 1: The false negative rate of Peddada's method and the one-stage ORICC algorithm**. (due to Liu et al.)

**Figure 4:**
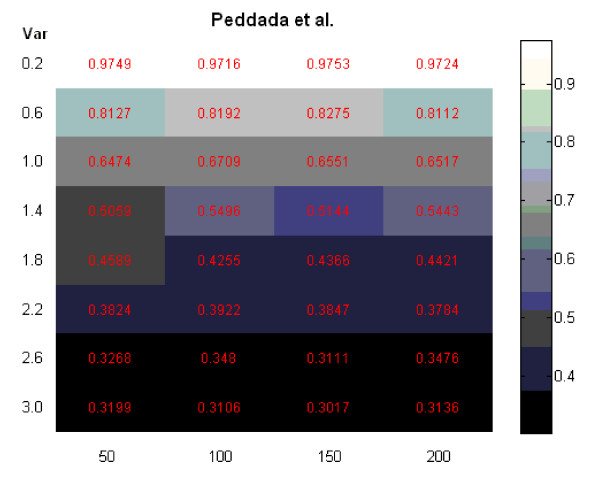
**Simulation 2: clustering precision of Peddada's method using a threshold of clustering 0.025**.

The second comment is that the false positive rates reported in Figure three in [7]were incorrect. We thank Peddada *et al*. for carefully reading our paper and pointing out this mistake. In our paper, we mistakenly stated the p-value threshold used for Peddada's method. The threshold was 0.5 instead of 0.025. We repeated all simulations in our paper involving Peddada's method using a p-value threshold of 0.025 and the results are presented in Figures 1, 2, 3 and 4 at the end of this report. Figures 1, 2 and 3 are for Simulation 1 in [7]and obtained by imposing the error rate for Peddada's method using threshold 0.025 on Figures 2, 3 and 4 in [7]. Figure 4 is for Simulation 2 in [7]and gives Rand's C statistics for the clusters given by Peddada's method using threshold 0.025. As pointed out by Peddada *et al*. in their correspondence, Peddada's method controls the false positive rate under the nominal level, i.e. the p-value threshold (See Figure 2). However, lower false positive rates are often at the price of increased false negative rates and also higher overall error rates (See Figures 1 and 3). Though a threshold of 0.5 seems unreasonable for p-values, it does offer an overall better clustering result than using 0.025 as the threshold. This is further confirmed by Rand's statistics in Figure 4. Except the comparison in false positive rates (Figure 2) is different from what we stated in our paper, other conclusions in our paper remain unchanged. It is worth noting that Peddada's method can achieve any false positive rate by using the corresponding p-value threshold.

In the situation of clustering microarray data, the clustering result often serves as a hypothesis generating tool, and gives the analysis a more exploratory flavor. Therefore, unlike in the classical hypothesis testing scenario, false negative and false positive are equally important in evaluating the clustering accuracy. Peddada *et al*. [8,9] treat the clustering problem more in a hypothesis testing way, therefore put more emphasis on controlling the false positive rate. Though our ORICC method is also based on order-restricted inference and has a similar structure to Peddada's method, we view the clustering problem as a model selection problem. By using a consistent model selection criterion, the false positive rate of our ORICC method approaches zero as the number of replicate arrays increases, whereas that of Peddada's method remains around the p-value threshold.

Peddada *et al*. also had two other comments on our inaccurate description of the optimality of the order-restricted MLE and the test used in Peddada's method. We appreciate their insight and agree that the description should be changed according to their suggestion.

Peddada *et al*. also reported a new simulation study based on a large number simulated null and non-null gene expressions, in which our one-stage ORICC algorithm was shown to have an inferior performance to the ORIOGEN algorithm. This motivated us to further explore the property of our ORICC algorithms. We repeated the same simulation with a varying number of null genes using ORIOGEN, one-stage ORICC and two-stage ORICC algorithms, and we found that, when null genes consist of the majority of all the genes, two-stage ORICC provides a much more satisfactory performance than one-stage ORICC, and has similar performance to ORIOGEN. See results in Table 2. It is worth noting that, in this simulation, the computational advantage of our algorithms remains, especially for the two-stage ORICC. For example, when there are 40000 null genes and 4000 non-null genes, the computational time is 128 minutes, 10.5 minutes and 3.0 minutes for ORIOGEN, one-stage ORICC and two-stage ORICC, respectively, on a workstation with a 2.30 GHz AMD Athlon(tm) 64 × 2 Dual Core 4400+processor and a 2.00 GB memory. Another issue worth mentioning is that ORIOGEN's performance depends on pre-specified p-value cutoff of 0.01 and reclassification p-value of 0.90. On a real data set, finding a proper choice of these cutoffs may be not easy.

**Table 2:** Comparison of ORIOGEN, one-stageORICC and two-stage ORICC. (due to Liu et al.)

Null gene	800	1000	2000	4000	16000	30000	40000
**ORIOGEN**							
Overall	0.2135	0.199	0.1735	0.1283	0.0579	0.0375	0.0310
Positive	0.0088	0.004	0.0085	0.0054	0.0107	0.0086	0.0077
Negative	0.1703	0.168	0.1773	0.1703	0.1685	0.1685	0.1788
**One-stage ORICC**							
Overall	0.1738	0.1726	0.2005	0.2218	0.2681	0.2863	0.2862
Positive	0.2863	0.3	0.3	0.2975	0.2994	0.3044	0.2995
Negative	0.0095	0.0075	0.0113	0.0103	0.0115	0.00975	0.0098
**Two-stage ORICC**							
Overall	0.2454	0.227	0.1968	0.1478	0.0607	0.0373	0.0303
Positive	0.005	0.004	0.0045	0.0023	0.0045	0.0033	0.0036
Negative	0.2183	0.212	0.2182	0.217	0.211	0.2163	0.217

To summarize, we thank Dr. Peddada and his colleagues for their insightful discussion, which motivated us to achieve a deeper understanding about the property of ORIOGEN, one-stage ORICC and two-stage ORICC. We hope that our work, their work and discussion, and this report will stimulate further work on clustering microarray data using order-restricted inference.

The authors are partly supported by the National Science Foundation of China (No.10871037).
